# Prevalence and impact of insomnia in children and adolescents with body dysmorphic disorder undergoing multimodal specialist treatment

**DOI:** 10.1007/s00787-019-01442-1

**Published:** 2019-11-23

**Authors:** Laura Sevilla-Cermeño, Daniel Rautio, Per Andrén, Maria Hillborg, Maria Silverberg-Morse, Guillermo Lahera, David Mataix-Cols, Lorena Fernández de la Cruz

**Affiliations:** 1 Karolinska Institutet, Department of Clinical Neuroscience, Child and Adolescent Psychiatry Research Center, Gävlegatan 22 (Entré B), Floor 8, 113 30 Stockholm, Sweden; 2 Stockholm Health Care Services, Stockholm County Council, Stockholm, Sweden; 3 Departamento de Medicina y Especialidades Médicas, Universidad de Alcalá, Madrid, Spain

**Keywords:** Body dysmorphic disorder, Insomnia, Cognitive-behavior therapy, Children, Adolescents

## Abstract

Pediatric body dysmorphic disorder (BDD) is challenging to treat. This study aimed to establish the prevalence of insomnia in youth with BDD and explore its impact on clinical outcomes. Sixty-six children and adolescents with BDD consecutively referred to a specialist clinic completed a range of clinical measures, including the Yale-Brown Obsessive–Compulsive Scale Modified for BDD-Adolescent Version (BDD-YBOCS-A), and the Insomnia Severity Index (ISI). Patients with clinical insomnia (ISI score ≥ 9) were compared to the rest of the sample on socio-demographic and clinical features. Fifty-six patients who received multimodal treatment were re-assessed post-treatment. A mixed-model ANOVA was performed to compare treatment outcomes between the insomnia vs. no insomnia groups, and Chi-squared tests were used to compare response and remission rates. According to the ISI, 48% of the sample qualified as having insomnia at baseline. These participants showed significantly higher self-reported BDD symptom severity, more depressive symptoms, and more functional impairment in daily activities. Patients with insomnia improved less on the BDD-YBOCS-A than those without insomnia, although the difference did not reach statistical significance. The rates of responders and remitters were lower in the insomnia group, compared to the non-insomnia group. Insomnia is prevalent in pediatric BDD, and is associated with more severe psychopathology and worse functioning in daily activities. Furthermore, youth experiencing BDD and insomnia may benefit from multimodal treatment to a lesser extent than those without insomnia. If these results are replicated in larger samples, treatment refinements for pediatric BDD could include specific modules to directly target insomnia.

**Electronic supplementary material:**

The online version of this article (10.1007/s00787-019-01442-1) contains supplementary material, which is available to authorized users.

## Introduction

Body Dysmorphic Disorder (BDD) is a psychiatric disorder characterized by a recurrent preoccupation with perceived defects in physical appearance that are not observable or appear minimal to others. Excessive repetitive behaviors (e.g., mirror checking) or mental acts (e.g., comparing own appearance with that of others) are often performed in response to the appearance concerns [1]. The estimated prevalence of BDD in both adult and adolescent community samples is approximately 2% [2, 3]. The onset of BDD is usually during adolescence [4, 5]. BDD in young people is associated with poor psychosocial functioning and low academic performance, with high rates of school abandonment [6, 7]. Furthermore, adolescents with BDD have been reported to have high levels of suicidality [4].

Cognitive-behavior therapy (CBT) is a moderately effective treatment for pediatric BDD. In one wait-list controlled trial, the between-group effect size was large (Cohen’s *d* = 1.13) at post-treatment and the response rate was 40% [8]. In the same trial, the effects of CBT were also durable over time (effect size = 1.70 at 12-month follow-up; response rate = 50%) [9]. CBT is recommended by clinical guidelines as the first-line treatment in children and adolescents with BDD [10]. If CBT is declined, not available, or the patient does not engage in it, a selective serotonin reuptake inhibitor (SSRI) may be considered [10]. However, while SSRIs are effective in adults with BDD, medication studies on pediatric patients are lacking. Importantly, although CBT is effective at the group level, a significant percentage of patients (46–60%) do not respond sufficiently to treatment [8, 11]. Hence, it is paramount to identify factors that could be hampering treatment adherence or response to CBT in patients with BDD to further improve outcomes in this vulnerable group.

Insomnia is a prevalent condition amongst youth [12, 13] and correlates with negative developmental outcomes, such as impaired emotional and behavioral regulation [14, 15]. The presence of insomnia has been linked to more severe psychopathology and worse general functioning in a variety of mental disorders in youth [16–18]. Additionally, reduced sleep has also been associated with impaired cognitive functioning, which may lead to impaired learning [19, 20]. As learning is a key aspect of CBT, poor sleep could potentially interfere with this treatment, reducing its overall efficacy. In other childhood psychiatric disorders, such as depression or bipolar disorder, there is solid evidence to indicate that sleep disturbances are associated with poorer treatment response and/or higher probabilities of relapse after remission [21, 22]. Conversely, interventions targeted at improving sleep have documented positive effects on various forms of psychopathology [23] and might even enhance the effects of CBT [24].

To our knowledge, to date, there have been no studies focusing on the prevalence of sleep problems in pediatric BDD and on whether these may interfere with evidence-based treatment. In an attempt to fill this gap in the literature, we report on one of the largest clinical samples of well-characterized youth with BDD, treated at a specialist obsessive–compulsive disorder (OCD) and related disorders clinic in Stockholm, Sweden. The aims of the study were threefold. First, we aimed to establish the prevalence of self-reported clinical insomnia in youth with BDD. Second, we aimed to compare the demographic and clinical characteristics of BDD patients with and without clinical insomnia. Finally, we aimed to explore whether insomnia had a negative effect on the response to multimodal treatment, including protocol-driven CBT and concurrent medication (when indicated), in this patient group.

## Methods

### Setting and participants

Participants were 66 children and adolescents meeting DSM-5 criteria for BDD [1] consecutively referred to a specialist pediatric OCD and related disorders outpatient clinic in Stockholm, Sweden, between January 2015 and March 2019. For a detailed description of the clinical setting, see Sevilla-Cermeño et al. [17]. The diagnoses were confirmed following a 3-h first assessment by a multidisciplinary clinical team, which included a full anamnesis and developmental history, full psychopathological screening with the Mini-International Neuropsychiatric Interview for Children and Adolescents (MINI-KID) [25], supplemented with additional modules for OCD and related disorders, and the Yale-Brown Obsessive–Compulsive Scale Modified for BDD-Adolescent Version (BDD-YBOCS-A) [26]. Socio-demographic information from the patients and their parents/caregivers was also gathered at this point. A series of self-reported measures covering a range of psychiatric conditions, including insomnia, were collected via an online platform (see “Measures” section below).

After the initial assessment, patients were offered treatment at our clinic, referred back to their local teams, or referred to more appropriate services elsewhere. Fifty-six (84.8%) of the sixty-six BDD patients received treatment for BDD at the clinic during the above-mentioned period.

All patients and their parents/legal guardians provided written consent to participate in the current study, which received ethical approval from the Regional Ethical Review Board in Stockholm (reference number 2015/1977-31/4).

### Measures

The Swedish language versions of the following measures were administered to all participants (*N* = 66) at baseline and, to the patients who received treatment at the clinic (*n* = 56), also at post-treatment.

#### Clinician-administered measures

The *BDD*-*YBOCS*-*A* is a clinician-administered semi-structured interview which is considered the gold-standard measure to rate the severity of BDD symptoms [26]. It comprises 12 Likert-type items ranging from 0 to 4: five severity items for both obsessions and compulsions and two further items to measure degree of insight and avoidant behavior. The total BDD severity score ranges from 0 to 48 [26]. The BDD-YBOCS-A has good psychometric properties, including strong internal consistency, good reliability, good convergent and discriminant validity, and sensitivity to change [27]. In our sample, the BDD-YBOCS-A had high internal consistency (Cronbach’s alpha = 0.84). As is standard in the field, *treatment response* was defined as a 30% or greater drop on the BDD-YBOCS-A from baseline to post-treatment, and *full or partial remission* as a total score ≤ 16 on the BDD-YBOCS-A at post-treatment [27, 28].

The *Clinical Global Impression*-*Severity* (CGI-S) is a single-item clinician-rated measure of symptom severity [29]. The item is rated on a seven-point scale. The CGI-S has demonstrated good concurrent validity and sensitivity to change [30].

The *Children´s Global Assessment Scale* (CGAS) is a single-item clinician-rated measure of the global functioning of a child or an adolescent during a specific period of time. Scores range from 1 (more disabled) to 100 (best functioning) and it has shown good psychometric properties with high reliability and both discriminant and concurrent validity [31].

All the above-mentioned clinician ratings were based on both the children and the parents report on the day of the assessment.

#### Self- or parent-administered measures

The *Insomnia Severity Index* (ISI) is a self-reported instrument measuring insomnia. It is composed of seven Likert-type items with a total score that ranges from 0 to 28 [32]. The Swedish translation of the ISI, which has been modified for use amongst youth, was used in this study [33]. This version has shown excellent psychometric properties with high internal consistency, construct validity, and a single factor structure in a clinical sample of pediatric patients with OCD [17]. The optimal cut-off score for detecting clinical insomnia in youths has been established to be 9 [34]. Using this established cut-off, our BDD sample was categorized into a clinical insomnia group (ISI scores ≥ 9) and a non-clinical insomnia group (ISI scores < 9).

The *Appearance Anxiety Inventory* (AAI) is a self-reported measure that focuses on the cognitive and behavioral processes that are characteristic to BDD [35]. It comprises ten items, with a total score ranging from 0 to 40, and includes two subscales: one measuring avoidance and the other measuring threat monitoring [35].

Depressive symptoms were assessed by the *Children’s Depression Inventory-Short Version* (CDI-S), a 10-item self-reported instrument examining the presence and severity of depressive symptoms in pediatric patients [36] and by the parent-reported version of the *Short Mood and Feeling Questionnaire (SMFQ*-*P),* a 13-item measure that evaluates mood in youths [37]. Both instruments have excellent psychometric properties.

The *Work, and Social Adjustment Scale-Youth Version* (WSAS-Y) is a short, self-reported instrument consisting of five items rated on a nine-point Likert scale. It assesses the degree of functional impairment in five areas, namely school, daily situations, social activities, leisure activities, and relationships [38]. The WSAS-Y is based on the original WSAS for adults [39] and has excellent psychometric properties [38]. The WSAS-Y also has a parent-rated version (WSAS-P) [38].

### Treatment

All patients treated at the specialist clinic (*n* = 56) received individual CBT delivered by clinical psychologists with extensive experience in the treatment of BDD cases. Of these, 32 were additionally prescribed medication for their BDD when deemed clinically appropriate (see Results section below). The CBT was protocol-driven, based on the manual developed by Mataix-Cols et al. for their trial [8]. Based on the experience in this adolescent BDD trial [8], the manual has undergone continued development by our team, in collaboration with the BDD team at the Maudsley Hospital, London. The updated version of the manual used in this study involves more therapy sessions than the original (typically up to 20 rather than 14 sessions). The developmentally tailored protocol is heavily based on exposure with response prevention (ERP) strategies. Since insight in this patient group tends to be low, the therapists try to promote motivation and engagement with ERP, often including motivational interviewing approaches to address the patients’ ambivalence. The treatment includes varying degrees of parental involvement, depending on the individual case formulation. In brief, the protocol consists of the following core elements: sessions 1 to 2–3 focus on psychoeducation about BDD and anxiety, perception, self-focused attention, and on the development of an ERP hierarchy; sessions 3–4 to 18 primarily focus on graded ERP (both therapist-assisted in vivo ERP and as homework assignments between sessions); and sessions 19 and 20 include strategies for relapse prevention and maintenance of the treatment gains. The protocol also includes optional modules, which are only used when required: these include mirror re-training, cognitive work, and attention re-training, amongst others (see Ref. [8]). Sessions last approximately 1 h and are usually conducted weekly, although complex patients are often offered more intensive approaches (e.g., several hours per day/week and home visits).

### Statistical analyses

SPSS version 25.0 for Windows software was used to analyze the data. Student’s *t* tests were used for between-group comparisons of continuous variables and Chi-squared tests for categorical variables. A mixed-model analysis of variance (ANOVA) was carried out to test for a differential effect of clinical insomnia on responsiveness to treatment. Response and remission rates for the two groups were compared using Chi-squared tests. All statistical tests were two-tailed. Statistical significance was set at *p *< 0.05.

## Results

### Participant characteristics at baseline

The sample (*N* = 66) consisted predominantly of girls (*n* = 56; 84.8%), with a mean age of 15.4 years (SD = 1.5; range 10–17) at baseline. The self-reported mean age of onset of their BDD was 12.9 years (SD = 1.7; range 8–17). The mean BDD-YBOCS-A score was 30.2 (SD = 5.0), reflecting moderate BDD symptom severity. A high proportion of the sample (*n* = 51; 77.3%) had a comorbid psychiatric disorder. The most common comorbid disorder was depression (*n* = 31; 47.0%).

### Prevalence of insomnia and characteristics of BDD patients with and without clinical insomnia at baseline

The mean ISI score for the sample was 9.2 (SD = 6.2). A total of 32 out of 66 participants with BDD (48.5%) scored above the established cut-off for clinical insomnia [34] at baseline. Table 1 shows the demographic and clinical characteristics of the groups with and without insomnia. Participants with clinical insomnia were slightly older at assessment than the participants without clinical insomnia (15.8 vs. 15.0 years, respectively; *p *= 0.034). Both groups were comparable in terms of gender, age at onset of BDD, family history of BDD, receipt of previous CBT, or prescription of any pharmacological treatment. The presence of comorbid depression was more frequent in the insomnia group, compared to those without insomnia (62.5% vs. 32.4%, respectively; *p *= 0.014), while the proportions of other comorbidities were not statistically different between groups.

**Table 1:** Comparison of demographic and clinical characteristics of patients with and without clinical insomnia at baseline (*N *= 66)

	Clinical insomnia (*n *= 32)		No clinical insomnia (*n *= 34)		Statistics	
	Mean	SD	Mean	SD	Student’s *t*	*p*
Age at assessment	15.8	1.2	15.0	1.7	− 2.16	0.034*
Age of BDD onset (*n* = 59)	13.2	1.5	12.6	1.9	− 1.27	0.211

	*N*	%	*N*	%	Chi-square	*p*
Gender						
Girls	30	93.8	26	76.5	4.01	0.135
Boys	2	6.3	7	20.6	4.01	0.135
Other	0	0.0	1	2.9	4.01	0.135
Family history of BDD	1	3.1	6	17.6	–	0.106^a^
Any comorbid mental disorder	26	81.3	25	73.5	0.56	0.454
Depression	20	62.5	11	32.4	6.02	0.014*
Anxiety disorders	6	18.8	12	35.3	2.28	0.131
OCD	8	25.0	4	11.8	1.94	0.164
ADHD	4	12.5	5	14.7	–	1.000^a^
ASD	4	12.5	4	11.8	–	1.000^a^
Eating disorders	1	3.1	2	5.9	–	1.000^a^
Tourette syndrome	1	3.1	0	0	–	0.485^a^
Hypochondriasis	0	0.0	1	2.9	–	1.000^a^
Previous CBT treatment	11	34.4	15	44.1	0.66	0.418
On pharmacological treatment	14	43.8	15	44.1	0.00	0.976
SSRI	10	31.3	11	32.4	0.01	0.923
Other Antidepressants	1	3.1	3	8.8	–	0.614^a^
Antipsychotics	1	3.1	2	5.9	–	1.000^a^
Antihistamines	6	18.8	4	11.8	–	0.505^a^
Melatonin	3	9.4	4	11.8	–	1.000^a^
ADHD medication	3	9.4	2	5.9	–	0.668^a^
Buspirone	0	0.0	3	8.8	–	0.239^a^

*ADHD* attention-deficit/hyperactivity disorder, *ASD* autism spectrum disorders, *OCD* obsessive–compulsive disorder, *SD* standard deviation, *SSRI* selective serotonin reuptake inhibitors

*Significant at 0.05; **significant at 0.01

^a^Fisher’s test

There were no between-group differences on the BDD-YBOCS-A or on other clinical measures, with the exception of the AAI, the WSAS-Y, and the WSAS-P (Table 2). Participants with clinical insomnia scored significantly higher on the AAI total score and the AAI avoidance subscale at baseline, indicating more severe self-reported BDD symptomatology. They also had higher scores on both WSAS scales, indicating worse self-reported and parent-reported functioning in daily activities. Specifically, BDD participants with insomnia scored significantly higher on the WSAS-Y item regarding family and other relationships, and on the WSAS-P item regarding everyday situations (Table 2).

**Table 2:** Comparison of clinical measures of patients with and without clinical insomnia at baseline (*N *= 66)

	Clinical insomnia (*n* = 32)		No clinical insomnia (*n* = 34)		Statistics	
	Mean	SD	Mean	SD	Student’s *t*	*p*
Insomnia measure						
ISI total	14.3	4.6	4.4	2.3	− 10.98	0.000**
BDD measures						
BDD-YBOCS-A total	30.3	5.0	30.2	5.1	− 0.06	0.953
BDD-YBOCS-A obsessions	13.0	2.4	12.8	2.3	− 0.41	0.687
BDD-YBOCS-A compulsions	12.6	2.5	12.9	2.3	− 0.54	0.593
AAI total (*n* = 63)	29.9	6.5	25.7	7.6	− 2.35	0.026*
AAI avoidance	19.7	4.1	16.1	5.4	− 3.02	0.004**
AAI threat monitoring	10.2	3.3	9.7	3.3	− 0.69	0.494
Other clinical measures						
CDI-S (*n* = 44)	11.9	4.3	10.6	4.7	− 0.95	0.351
SMFQ-P	15.6	6.3	15.1	5.9	− 0.38	0.708
CGI-S	4.8	0.6	4.7	0.6	− 1.04	0.302
CGAS	45.3	7.7	47.3	4.6	1.30	0.199
WSAS-Y total	23.2	6.9	18.9	4.9	− 2.94	0.005**
WSAS-Y school	6.3	1.5	5.7	1.8	− 1.50	0.138
WSAS-Y everyday situations	3.4	2.6	2.3	1.9	− 1.87	0.066
WSAS-Y social activities	5.8	2.0	5.4	1.8	− 0.86	0.396
WSAS-Y leisure time	2.5	2.4	1.6	1.9	− 1.66	0.102
WSAS-Y family and relationships	5.2	2.0	3.9	2.1	− 2.69	0.009**
WSAS-P total	23.8	7.0	20.3	6.4	− 2.10	0.040*
WSAS-P school	6.6	1.6	6.1	2.2	− 1.19	0.237
WSAS-P everyday situations	4.1	2.1	3.0	2.2	− 2.05	0.045*
WSAS-P social activities	5.6	2.1	5.6	2.2	− 0.01	0.994
WSAS-P leisure time	2.6	2.2	1.6	2.2	− 1.73	0.088
WSAS-P family and relationships	5.0	2.3	4.1	2.3	− 1.50	0.131

*AAI* Appearance Anxiety Inventory, *BDD-YBOCS* Yale-Brown Obsessive–Compulsive Scale Modified for BDD-Adolescent version, *CDI-S* Children’s Depression Inventory-Short Version, *CGAS* Children’s Global Assessment Scale, *CGI-S* Clinical Global Impression-Severity, *ISI* Insomnia Severity Index, *SMFQ-P* Short Mood and Feeling Questionnaire, *WSAS-Y* Work, Social and Adjustment Scale-Youth Version, *WSAS-P* Work, Social and Adjustment Scale-Parent Version

*Significant at 0.05; **significant at 0.01

### Treatment characteristics of BDD patients with and without insomnia

A total of 56 patients underwent CBT treatment at the clinic. There were no significant differences on any baseline clinical measures between those participants who were treated at the clinic and those who were not (Supplementary Table 1). The mean number of sessions received was 17.3 (SD = 12.4; range 4–80). Twenty-four of the treated patients (42.9%) scored above the ISI cut-off for clinical insomnia. There were no significant differences between the insomnia (*n* = 24) and the non-insomnia (*n* = 32) groups in terms of the total number of CBT sessions received (16.5 vs. 17.9 sessions, respectively; Student’s *t *= 0.41, *p *= 0.687).

Thirty-two (57.1%) of the participants received medication for their BDD alongside the CBT treatment. Of those, 26 patients received treatment with an SSRI, one patient with an antipsychotic (aripiprazole), and five patients received an SSRI in combination with an antipsychotic (aripiprazole in all five cases). There were no differences in BDD symptom severity between those receiving vs. those not receiving medication (30.7 vs. 30.3 on the BDD-YBOCS-A, respectively; Student’s *t *= –0.31, *p *= 0.762). Similarly, the comparison between the percentage of patients on medication in the insomnia group (54.2%) and in the non-insomnia group (59.4%) did not reveal significant differences (Chi-square = 0.15, *p *= 0.697).

Additionally, 15 of the 56 treated participants (26.8%) received medication for insomnia. Of those, nine were prescribed melatonin, four received antihistamines as hypnotics, and two patients took melatonin in combination with an antihistamine. Patients receiving hypnotic medication showed equally severe BDD symptoms at baseline when compared to the ones who did not (31.7 vs 30.1 on the BDD-YBOCS-A, respectively; Student’s *t *= –1.01, *p *= 0.296). Of note, the percentage of patients who received any hypnotic drugs in the insomnia group (29.2%) and in the non-insomnia group (25%) was not significantly different (Chi-square = 0.12, *p *= 0.728).

### Treatment outcomes in BDD patients with and without clinical insomnia

Scores for the ISI, the BDD-YBOCS-A, the AAI, and other clinical measures in the groups with and without clinical insomnia at baseline and post-treatment are shown in Tables 3 and 4.

**Table 3:** Insomnia- and body dysmorphic disorder-specific severity scores at baseline and post-treatment, by insomnia status (*N *= 56)

	Clinical insomnia (*n *= 24)		No clinical insomnia (*n *= 32)		Statistic *t* test	*p*
	Mean	SD	Mean	SD		
ISI total						
Baseline	14.5	4.8	4.4	2.4	− 9.52	0.000**
Post-treatment	11.2	6.6	3.5	4.5	− 4.24	0.000**
BDD-YBOCS total						
Baseline	31.2	5.0	30.1	5.3	− 0.79	0.432
Post-treatment	15.5	9.6	11.3	6.9	− 1.91	0.062
BDD-YBOCS obsessions						
Baseline	13.3	2.3	12.8	2.3	− 0.87	0.390
Post-treatment	6.9	4.0	5.3	3.0	− 1.75	0.086
BDD-YBOCS compulsions						
Baseline	13.0	2.7	12.8	2.4	− 0.23	0.820
Post-treatment	6.4	4.6	4.6	3.3	− 1.64	0.110
AAI Total						
Baseline	30.5	6.7	25.5	7.6	− 2.46	0.017*
Post-treatment	17.4	12.2	12.0	8.7	− 1.71	0.095
AAI avoidance						
Baseline	19.9	3.9	15.9	5.4	− 3.12	0.003**
Post-treatment	10.8	7.8	8.0	5.7	− 1.39	0.172
AAI threat monitoring						
Baseline	10.6	3.5	9.7	3.4	− 1.00	0.324
Post-treatment	6.6	4.6	4.0	3.6	− 2.06	0.046*

*AAI* Appearance Anxiety Inventory, *BDD-YBOCS-A* Yale-Brown Obsessive–Compulsive Scale Modified for BDD-Adolescent version, *ISI* Insomnia Severity Index, *SD* standard deviation

*Significant at 0.05; **significant at 0.01

**Table 4:** Depression and general functioning scores at baseline and post-treatment, by insomnia status (*N *= 56)

	Clinical insomnia (*n *= 24)		No clinical insomnia (*n *= 32)		Statistic *t* test	*p*
	Mean	SD	Mean	SD		
CDI-S						
Baseline	11.9	4.5	10.6	4.7	− 0.89	0.377
Post-treatment	8.6	5.3	6.0	4.3	− 1.75	0.087
SMFQ-P						
Baseline	15.7	6.2	15.5	5.8	− 0.13	0.898
Post-treatment	10.7	7.7	8.4	5.9	− 1.16	0.254
CGI-S						
Baseline	4.8	0.7	4.6	0.6	− 1.36	0.180
Post-treatment	3.0	1.4	2.0	1.1	− 2.86	0.006**
CGAS						
Baseline	43.8	7.7	47.2	4.7	2.02	0.048*
Post-treatment	52.9	7.4	59.6	9.7	2.40	0.020*
WSAS-Y						
Baseline	24.1	6.2	19.0	5.0	− 3.41	0.001**
Post-treatment	13.6	7.4	8.8	6.2	− 2.32	0.025*
WSAS-P						
Baseline	23.7	7.1	20.4	6.5	− 1.81	0.075
Post-treatment	16.8	8.0	11.9	7.4	− 2.13	0.038*

*CDI-S* Children’s Depression Inventory-Short Version, *CGAS* Children’s Global Assessment Scale, *CGI-S* Clinical Global Impression-Severity, *SMFQ-P* Short Mood and Feeling Questionnaire, *WSAS-Y* Work, Social and Adjustment Scale-Youth Version, *WSAS-P* Work, Social and Adjustment Scale-Parent Version

*Significant at 0.05; **significant at 0.01

**Fig. 1:**
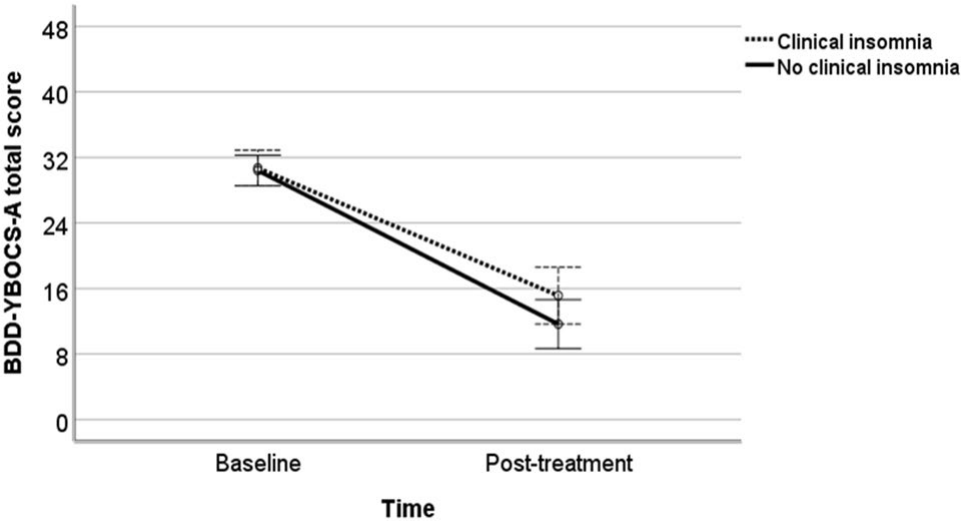
Mean Yale-Brown Obsessive–Compulsive Scale Modified for BDD-Adolescent version (BDD-YBOCS) total scores at baseline and post-treatment, by insomnia status (baseline depression status—coded as present/absent—and age at assessment are included as covariates in the model)

A mixed-model ANOVA with a within-subjects factor of time (baseline vs. post-treatment) and a between-subjects factor of group (with vs. without clinical insomnia) was performed. Because the groups differed at baseline regarding the presence of comorbid depression and the mean age at the time of assessment, we added comorbid depression (coded as present/absent) and age at assessment as covariates in the model. The model revealed a main effect of time (*F* [1, 52] = 328.77, *p *< 0.001), indicating a significant reduction in the BDD-YBOCS-A total score over the course of the treatment. There was no significant main effect of group (*F* [1, 52] = 0.59, *p *= 0.446) or time-by-group interaction (*F* [1, 52] = 2. 38, *p *= 0.129) (Fig. 1).

Given that we found differences in baseline self-reported BDD severity scores between groups, we repeated the same model using the AAI total score as the outcome variable. Results revealed a significant effect of time (*F* [1, 39] = 68.50, *p *< 0.001), as well as a significant group effect (*F* [1, 39] = 4.60, *p *= 0.038), indicating that the insomnia group had more severe self-reported BDD symptoms through treatment, but, mirroring the results of the analysis using the BDD-YBOCS-A, no significant time-by-group interaction effects were found (F [1, 39] = 0.12, *p *= 0.733).

A final model revealed that symptoms of clinical insomnia, as measured by the ISI, also improved during the course of the treatment in the whole, treated sample (*F* [1, 42] = 6.72, *p *= 0.013) (Table 3).

**Fig. 2:**
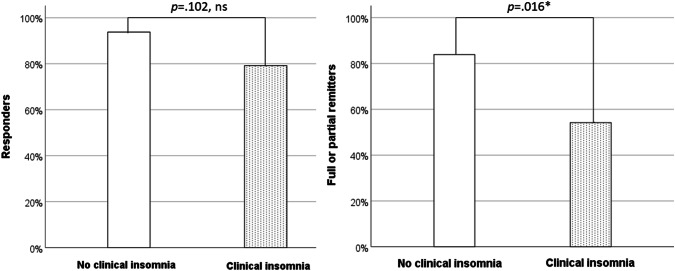
Rates of responders and full or partial remitters, by insomnia status

The mean percentage decrease in BDD symptom severity from baseline to post-treatment, measured by the BDD-YBOCS-A, was 51.6% in the clinical insomnia group and 62.8% in the non-clinical insomnia group (Student’s *t *= 1.82, *p *= 0.074). The proportion of treatment responders in the insomnia group was lower (19/24, 79.2%) than the proportion of treatment responders in the non-insomnia group (30/32, 93.8%), but this difference did not reach statistical significance (Chi-square = 2.67, *p *= 0.102). However, the proportion of patients classed as being in full or partial remission at post-treatment was significantly lower in the insomnia group when compared to the non-insomnia group (13/24, 54.2% vs. 26/31, 83.9%, respectively; Chi-square = 5.79, *p *= 0.016) (Fig. 2).

## Discussion

To our knowledge, the present study is the first to explore the prevalence of clinical insomnia in a large sample of well-characterized youth with BDD. We also examined the influence of insomnia on the clinical features and response to multimodal treatment in a subsample of treated patients. The study had three main findings.

Our first and main finding was that, in our sample, about half of the participants experienced significant insomnia according to the ISI, a well-validated insomnia measure. Furthermore, the ISI mean score for the whole group was slightly above the established cut-off for clinical insomnia [34]. The observed prevalence (48.5%) and overall severity of insomnia in this sample (a mean ISI score of 9.2) were both higher than those reported in a pediatric OCD sample of patients recruited from the same clinic (prevalence of 42% and mean ISI score of 7.6) [17], which is worth noting given the described similarities between the two disorders. These results suggest that insomnia is common and should be assessed and managed in young people with BDD.

Second, young people who experienced clinical insomnia scored higher on self-reported measures of BDD symptom severity, particularly avoidance behavior, had higher rates of comorbid depression, and also worse functioning in daily activities, when compared to patients without insomnia. Regarding BDD symptom severity at baseline, it is worth noting that no between-group differences were found on the clinician-rated BDD-YBOCS-A. The AAI and the BDD-YBOCS-A measure related constructs, but their content is only partially overlapping. In the original validation study, the correlation between these instruments was in the region of 0.5 [35]. In our study, the inter-correlation between these scales was 0.3. Notably, while the AAI has a separate avoidance scale, the BDD-YBOCS-A incorporates avoidance it its total score. Thus, our results might indicate that insomnia is most likely associated with the severity of avoidance in BDD.

Pediatric BDD patients with insomnia also had higher rates of comorbid depression than those without insomnia. This is somewhat expected given the known association between depression and sleep disorders [16], and the fact that insomnia and/or hypersomnia are listed as diagnostic criteria for depression [1]. Interestingly, however, no between-group differences were found in the self-reported or parent-reported measures of depressive symptoms. The fact that none of these instruments include sleep items could explain the lack of differences between groups. Thus, it is possible that individuals with insomnia in our sample may not be more depressed than those without, but be more likely to be diagnosed with depression, simply because insomnia is one of the diagnostic criteria for the disorder.

The BDD and insomnia group also had worse functioning in daily activities like family relationships, social, and everyday situations. The link between insomnia and worse functioning has also been previously reported in a variety of other psychiatric conditions in children and adolescents like OCD [17], anxiety disorders [40], autism spectrum disorders [41], and attention-deficit/hyperactivity disorder [42].

Third, we found suggestive evidence that insomnia may have a negative impact on treatment outcomes, although the results should be taken as preliminary until replicated in larger samples. Our statistical model including insomnia status as a between-group factor and adjusting for comorbid depression and age at assessment did not show significant time-by-group interactions, implying that both groups, with and without insomnia, improved similarly. However, the proportion of responders and remitters after completing treatment was substantially lower in the insomnia group, when compared to the non-insomnia group. If confirmed, this may have important clinical implications as not reaching remission status after treatment has been associated with higher odds of relapse in related disorders like OCD [43]. It is worth noting that our study was conducted in a naturalistic setting where patients with self-reported insomnia problems may have received additional support during the course of the multimodal treatment (e.g., sleep hygiene instructions and hypnotic medication). Those additional interventions may have partially masked the impairment caused by the sleep problems on the patients’ functioning and their treatment outcomes. Future studies using larger samples of BDD cases are warranted to elucidate the potential impact of insomnia on treatment response. If confirmed, the association between insomnia and worse clinical outcomes may suggest that current treatment protocols could be refined further to include specific insomnia modules.

Another intriguing implication for future research is that insomnia-specific interventions may, by themselves, have a beneficial effect on BDD symptoms without necessarily directly targeting the appearance concerns [23]. Interestingly, in our sample, insomnia symptoms also improved significantly after treatment. This finding is in line with the previous research in pediatric depression [44] and in pediatric OCD [17], showing that treatments targeting psychiatric symptoms also tend to improve sleep.

This study has some limitations. First, our sample size was modest and our analysis of the outcome data may have been underpowered to detect significant differences. The results will require replication and extension in larger patient cohorts. Second, we used a self-reported measure of insomnia rather than a structured diagnostic interview. However, the ISI cut-off employed in this study has been well validated against diagnosed insomnia cases [32]. Nevertheless, further studies including parent-reported and objective measures of insomnia are warranted. Third, this study was conducted in a specialist setting which receives referrals for relatively severe and/or complex cases, and hence, the findings may not be generalizable to other, milder populations of BDD cases. However, the clinical characteristics of our sample resemble those of other clinical BDD samples from around the world. Finally, follow-up data beyond the end of the treatment were not available for this study. It will be relevant for future studies to examine the possible influence of insomnia over the maintenance of treatment gains in the long run.

## Conclusions

Insomnia is prevalent in pediatric BDD, and is associated with higher scores on self-reported measures of BDD symptom severity, higher rates of comorbid depression, and worse functioning in daily activities. Young BDD patients experiencing insomnia improved with multimodal treatment, but to a lesser extent than BDD patients without insomnia. If these results are replicated in larger samples, treatment refinements for pediatric BDD could include specific modules to directly target insomnia.

## Electronic supplementary material

Below is the link to the electronic supplementary material.

Supplementary material 1 (DOCX 14 kb)
