# Supplementary material for: Prevalence and impact of insomnia in children and adolescents with body dysmorphic disorder undergoing multimodal specialist treatment
Source: Eur Child Adolesc Psychiatry. 2019 Nov 23;29(9):1289–99. doi: 10.1007/s00787-019-01442-1 (PMC7497371; doi:10.1007/s00787-019-01442-1)
Supplement: Supplementary file 1 — Supplementary material 1 (DOCX 14 kb) [file 787_2019_1442_MOESM1_ESM.docx]

**SUPLEMENTARY MATERIAL**

**Supplementary Table 1.** Comparison of clinical measures of patients who received treatment and patients who did not (*N*=66).

|  | **Treated patients** | | **Untreated patients** | | **Statistics** | |
| --- | --- | --- | --- | --- | --- | --- |
|  | **(n=56)** | | **(*n=*10)** | |  |  |
| **INSOMNIA MEASURE** | **Mean** | **SD** | **Mean** | **SD** | **Student´s *t*** | ***p*** |
| ISI Total | 8.7 | 6.2 | 11.9 | 5.7 | 1.53 | .131 |
|  |  |  |  |  |  |  |
| **BDD MEASURES** | **Mean** | **SD** | **Mean** | **SD** | **Student´s *t*** | ***p*** |
| BDD-YBOCS-A Total | 30.5 | 5.1 | 28.4 | 4.0 | -1.25 | .217 |
| BDD-YBOCS-A Obsessions | 13.0 | 2.3 | 12.3 | 2.6 | -.85 | .401 |
| BDD-YBOCS-A Compulsions | 12.9 | 2.5 | 11.9 | 1.5 | -1.24 | .221 |
| AAI Total (n=63) | 27.7 | 7.6 | 28.3 | 6.1 | .24 | .808 |
| AAI Avoidance | 17.6 | 5.2 | 19.1 | 4.6 | .85 | .397 |
| AAI Threat Monitoring | 10.1 | 3.4 | 9.2 | 2.3 | -.78 | .441 |
|  |  |  |  |  |  |  |
| **OTHER CLINICAL MEASURES** | **Mean** | **SD** | **Mean** | **SD** | **Student´s *t*** | ***p*** |
| CDI-S (n=44) | 11.2 | 4.6 | 10.8 | 3.9 | -.24 | .813 |
| SMFQ-P | 15.6 | 5.9 | 13.9 | 7.1 | -.81 | .423 |
| CGI-S | 4.7 | 0.7 | 4.9 | 0.6 | .92 | .362 |
| CGAS | 45.7 | 6.3 | 49.6 | 5.4 | 1.82 | .073 |
| WSAS-Y Total | 21.2 | 6.1 | 19.9 | 7.1 | -.58 | .564 |
| WSAS-P Total | 21.8 | 6.9 | 23.0 | 7.1 | .50 | .621 |

*Note:* * Significant at 0.05; ** significant at 0.01.

*Abbreviations:* AAI, Appearance Anxiety Inventory; BDD-YBOCS-A, Yale-Brown Obsessive-Compulsive Scale Modified for BDD–Adolescent version; CDI-S, Children’s Depression Inventory – Short Version; CGAS, Children´s Global Assessment Scale; CGI-S, Clinical Global Impression – Severity; SMFQ-P, Short Mood and Feeling Questionnaire; WSAS-Y, Work, Social and Adjustment Scale – Youth Version; WSAS-P, Work, Social and Adjustment Scale – Parent Version. ISI, Insomnia Severity Index; SD, standard deviation.
